# Double Crush Syndrome of the L5 Nerve Root and Common Peroneal Nerve at the Fibular Head: A Case Series and Review of the Literature

**DOI:** 10.3390/jcm14145023

**Published:** 2025-07-16

**Authors:** Hugo F. den Boogert, Janneke Schuuring, Godard C. W. de Ruiter

**Affiliations:** 1 Department of Neurosurgery, Haaglanden Medical Center, 2512 VA The Hague, The Netherlands; 2 Department of Neurology, Groene Hart Hospital, 2803 HH Gouda, The Netherlands

**Keywords:** double crush syndrome, lower extremity, L5 nerve root, peroneal nerve, compression neuropathy, peroneal decompression

## Abstract

**Background/Objectives**: The co-existence of multiple compression sites on the same nerve can pose a clinical and diagnostic challenge, warranting a different treatment strategy. This so-called double crush syndrome (DCS) has mainly been investigated in the upper limb. Only a few studies have investigated DCS for the lower limb. In this article, a single-center illustrative clinical case series is presented, and current literature on L5 nerve root (NR) and concomitant common peroneal nerve (CPN) is reviewed. **Methods**: All patients presenting between 2019 and 2022 with L5 nerve root (NR) compression and, along their clinical courses, concomitant compression of the common peroneal nerve (CPN) at the fibular head were included. Information on clinical features, diagnostics and surgeries was obtained. The outcome was assessed at the last outpatient follow-up appointment. In addition, an extensive literature review has been conducted. **Results**: Fourteen patients were included with a mean follow-up of 6.8 months. The majority had pain (71%) or motor deficits (71%). Seven patients were referred for clinical and radiological L5 NR compression but were also found to have CPN compression; the other seven patients had persisting or recurrent symptoms after surgically or conservatively treated L5 NR compression, suggestive of additional peroneal neuropathy. All patients had CPN decompression at the fibular head, with successful results obtained in 93% of the patients. Pain of the lower leg improved in all patients, and dorsiflexion function improved in 78%. **Conclusions**: Concomitant L5 NR and CPN appear to occur more frequently than expected. Peroneal neuropathy can present simultaneously with L5 nerve radiculopathy or after surgically or conservatively treated L5 NR compression. Overlapping symptoms and variation in clinical presentations make it difficult to diagnose and, therefore, underrecognized. More awareness among treating physicians of this specific double crush syndrome is important to prevent any delay in treatment, in this case, a less invasive common peroneal nerve release at the fibular head, and to avoid unnecessary (additional) spinal surgery.

## 1. Introduction

Compression of the L5 nerve root (NR) due to degenerative causes is frequently seen in spine clinics, presenting with classical pain starting in the lower back, radiating over the ventrolateral side of the leg to the dorsum of the foot and toes [1,2]. Distally, the L5 nerve branches off to the common peroneal nerve (CPN, also called the common fibular nerve). Entrapment of the CPN at the level of the fibular head, before it divides into the deep and superficial branches, is one of the most common neuropathies of the lower extremity, possibly because of its relatively superficial location and close relation with the fibular prominence and the overlying fascia and intermuscular septa of the peroneal muscles [3,4,5]. It causes the same radiating pattern starting from the lateral side of the knee, and similar to L5 radiculopathy, it can be accompanied by loss of sensation and reduced dorsiflexion of the foot [5,6,7,8]. The clinical overlap can make it difficult to distinguish peroneal neuropathy from L5 radiculopathy, especially if the patient also has low back pain. It can be even more clinically challenging if these pathologies co-exist, a so-called double crush syndrome.

**Figure 1:**
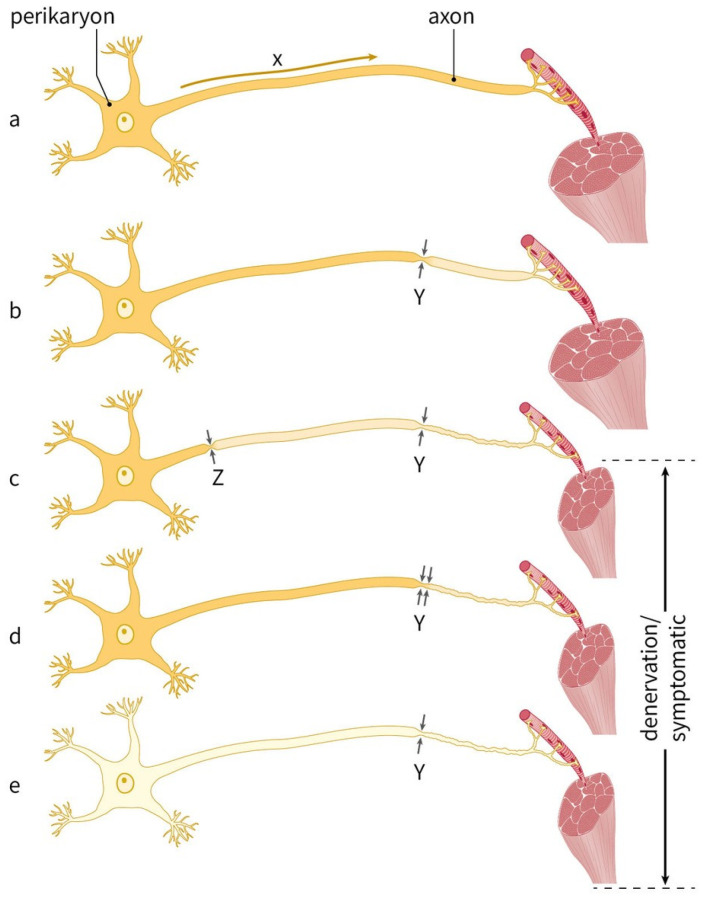
Concept of double crush recreated (modified from the original image by Upton and McComas [9]). (**a**) Describing the normal situation with axoplasmic flow (x) inside a single axon. (**b**) A mild compression (y) does not lead to significant disruption of flow or otherwise significant injury, leading to denervation. (**c**) An additional mild (z) compression site. Either the cumulative reduction in axoplasmic flow below safety margins [9] or other proposed pathomechanisms for dual lesion neuropathy [30] results in denervation and subsequent symptoms. Displaced in the figure is a single axon. Considering that the peripheral nerve has a large number of axons, one can also argue that a mild compression proximal only affects a part of the axons, whereas an additional distal (also mild) compression site affects a different portion, resulting in a combined significant loss of nerve conduction and subsequent clinical symptoms (**d**,**e**) Denervation also occurs due to one severe compression site (y) or a mild compression in an already damaged axon, for example, in diabetes patients (**e**).

Double crush syndrome (DCS) was first described in 1973 to explain the findings of co-existent proximal cervical root lesions and distal ulnar or median neuropathy [9]. Equivalent to one severe compression site, the combination of two co-existing mild compression sites can result in significant denervation and subsequent symptoms (Figure 1). Over the past decades, animal models and clinical data have provided support for this theory [10,11,12], and although the occurrence of DCS seems to be recognized by a wide variety of physicians [13], controversy remains [14,15,16]. In contrast to clinical studies on DCS of the upper limbs [17,18,19], reports on DCS of the lower extremities are scarce [20,21,22,23,24,25,26,27,28,29].

The goal of this article is to describe the different patterns of co-occurrence of L5 radiculopathy and CPN compression at the fibular head in a single surgeon’s practice and report on the typical signs suggestive of concomitant pathologies, the diagnostic modalities that can help to differentiate between them and the outcomes of the different surgical strategies. In addition, we reviewed current literature to achieve a better understanding of the occurrence of this specific double crush phenomenon.

## 2. Materials and Methods

### 2.1. Patient Selection and Data Collection

Patients presented to the senior author (G.d.R.), specialized in both peripheral nerve and spine surgery, between May 2019 and June 2022 with along their clinical course (suspicion of) co-existing L5 NR compression and CPN entrapment at the fibular head, were included in this study. Standard care was given, and all surgical procedures were performed by the senior author. The study was approved by a local ethics committee.

Demographics, clinical features, diagnostic pre-operative workup and surgical data were obtained. This included the patient’s history with emphasis on patterns of pain irradiation in the leg, presence of Tinel’s sign, presence of altered sensation and loss of strength reported on a standard Medical Research Council scale (MRC grades 0 to 5) [31]. Ultrasound (US) is preferred in our hospital over nerve conduction studies (NCS) and electromyography (EMG) for detecting CPN entrapment at the fibular head. According to nationwide protocols, our hospital wields a cut-off value of an abnormal cross-sectional area (CSA) of the peroneal nerve of >11 mm^2^ at the level of the fibular head using ultrasound as an imaging modality [32]. If measured, the CSA of the asymptomatic contralateral common peroneal nerve was also reported. In some cases where additional NCS/EMG was indicated, or patients were referred with NCS/EMG, this is reported as well. Both NCS/EMG and US were performed in a standardized manner by a team experienced with this pathology.

### 2.2. Follow Up

All patients underwent decompression of the CPN at the fibular head. The first outpatient clinic follow-up visits were at 6 weeks after surgery. If symptoms had not improved, additional follow-up was scheduled at 3 months after the surgery. Additional diagnostic testing was performed if symptoms persisted. The primary outcome was measured at the latest outpatient clinic follow-up appointment. A subjective outcome measurement, as graded by the patient, was noted using a 5-point Likert scale [33]. Successful relief of symptoms (e.g., pain, paresthesia, numbness or loss of strength) was defined as Likert 1 (complete) and Likert 2 (almost complete recovery). Partial relief (Likert 3) was not considered a successful result. Likert 4 (no change in symptoms) and Likert 5 (worsened symptoms) were defined as failures. In addition, the effect of the procedure on pain/paresthesia and loss of strength was separately measured.

### 2.3. Surgical Technique of Decompression of the Common Peroneal Nerve

Patients were operated on in a supine position under general anesthesia with the leg in almost 90 degrees of flexion and the knee slightly turned medially. A proximal tourniquet was used. A small, curved incision was made around the fibular head. The layers of superficial fascia were divided more proximally to expose the CPN. The nerve was then carefully dissected further distally until it coursed underneath the peroneus longus muscle (PLM). The overlying fascia of the peroneus longus muscle was cut, and the muscle retracted in order to identify and cut the posterior intermuscular septum, usually the site of entrapment [5,7]. Patients were discharged the same day.

### 2.4. Review of the Current Literature

PubMed and EMBASE were searched up to April 2025 using the keywords “double crush”, “concomitant nerve injury”, “L5 nerve root compression”, “common peroneal nerve compression”, “fibular nerve”, and their synonyms. Only clinical studies reporting on potential concomitant L5 nerve root compression and distal peroneal neuropathy were included. Studies in other languages than English or Dutch, or lacking full text, were excluded. Included articles were screened for additional eligible references.

### 2.5. Statistical Analysis

Data was recorded in Castor Clinical Data Management System (CDMS, v2024.3.4.0). Continuous data were reported as mean ± standard deviation or range. If applicable, data on demographics, clinical features and diagnostic findings were categorized and reported as the number and percentage of the total number of patients. Due to the small sample, no further statistical analysis has been performed.

## 3. Results

### 3.1. Case Series

#### 3.1.1. Clinical Characteristics

Fourteen patients were included in this study; their clinical data are presented in Table 1. The mean age was 57 years (range 42–72 years), and most of them were men (N = 10, 71%). The majority of patients had motor deficit (71%) and/or radiating pain (71%); less than half (43%) also reported numbness of the lower leg. The average MRC grade of dorsiflexion among patients with motor deficits was 3.6 (range 1–4). Twelve patients (86%) reported back pain at initial presentation, and Tinel’s sign of the CPN at the fibular head was positive in 29% of the cases. All patients had pre-operative MRI of the spine and US of the CPN at the fibular head; additional NCS/EMG recordings had been performed in 9 patients (64%). MRI showed a degenerative lateral recess stenosis in six patients (43%), foraminal L5 stenosis (with or without spondylolisthesis) in five patients (36%), and a herniated disk with L5 compression in three patients (21%). The mean CSA of the symptomatic CPN was 16.6 mm^2^ (range 11–37 mm^2^). In six patients, the contralateral asymptomatic peroneal nerve was also measured with a mean CSA of 13.3 (range 12.0–15.9).

#### 3.1.2. Treatment and Outcome

The average follow-up after CPN decompression at the fibular head was 6.8 months (range 1.5–14.5 months). Despite substantial clinical variety among cases, two distinct groups could be identified. The first group of seven patients (50%) were referred for spinal surgery with symptoms and radiological findings fitting L5 NR pathology, but based on history and neurologic examination, concurrent CPN entrapment was suspected. In this group, over 70% of the patients reported having radiating leg pain; however, in the majority of cases (four out of five patients), the symptoms were predominantly localized in the lower leg, which raised the suspicion of an additional peroneal neuropathy. This was confirmed by US or NCS/EMG, and neurolysis of the CPN was performed. In this group, only one patient needed additional spinal surgery two months later due to progressive symptoms (no 7 in Table 1). The second group, consisting of the other seven patients, had been surgically (N = 5) or conservatively treated (N = 2) for L5 NR compression but had persisting or recurrent symptoms suggestive of concomitant peroneal neuropathy. All patients in this group presented with back pain, and 86% had radiating symptoms in the entire leg. Upper leg symptoms and back pain improved after spinal surgery, but symptoms of the lower leg remained, usually numbness or a new onset of a footdrop. Two patients reported new lower leg complaints 3 and 14 months after surgery; the other three patients experienced symptoms directly after surgery. If those symptoms were new or persisting was not always clear due to the masking effect of the heavy radiating pain before surgery.

Overall, in 13 patients (93%), successful results were obtained after CPN decompression, defined as complete recovery (Likert 1; 4 patients, 28%) or almost complete recovery (Likert 2; 9 patients, 64%). One patient had only partial recovery (Likert 3) at his last follow-up of 11 months but was at that point steadily improving. He did not attend his next follow-up appointment. No patients worsened after surgery (Likert 5). Of the 10 patients with pre-operatively pain or paresthesia, all patients reported improvement at the latest follow-up. For the 10 patients with pre-operative loss of strength of dorsiflexion and/or eversion of the foot, 7 (70%) showed improvement with an average MRC grade of 4.4 (range 2–5), compared to 3.6 (range 1–4) pre-operatively.

### 3.2. Literature Review

**Figure 2:**
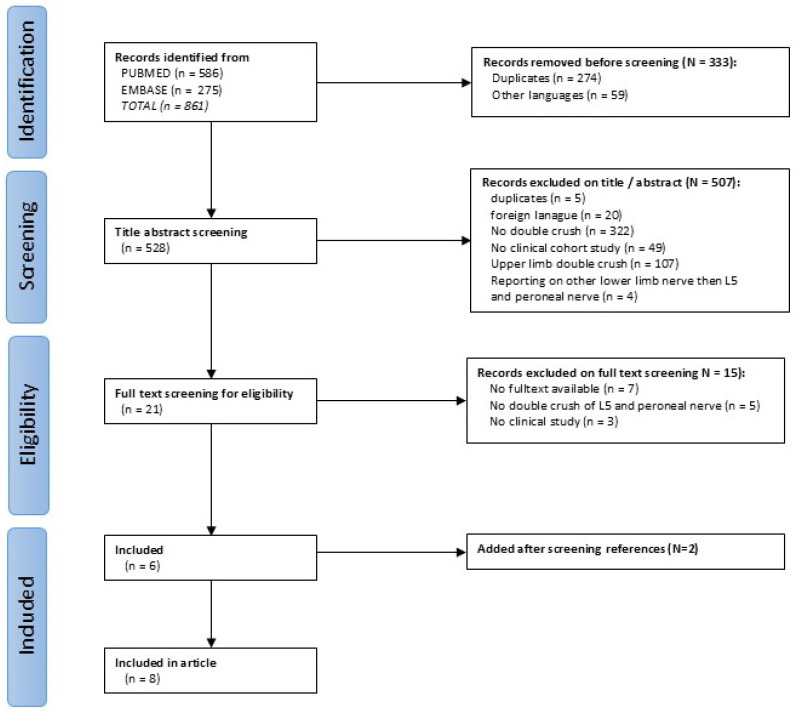
Screening according to the Prisma Guidelines [34].

After screening a total of 528 articles, 21 articles were selected for full-text evaluation. Six articles were eligible for inclusion; after screening the references, another two articles were added (Figure 2). Of the eight included studies, three were retrospective case series [20,21,29], and the rest were case reports [21,22,23,24,25]. The results of the review are shown in Table 2.

## 4. Discussion

In this article, we describe an illustrative clinical case series of fourteen patients with, along their clinical course, concomitant L5 nerve radiculopathy and CPN entrapment at the fibular head, treated at a high-volume spinal and peripheral nerve center. We found that decompression of the CPN in these patients with this specific type of double crush injury leads to complete or almost complete improvement of symptoms (e.g., pain, tingling sensation, numbness) in 93% of the cases. Specifically, radiating pain was reduced in all patients, and improvement of dorsiflexion function was seen in 70%. A comprehensive literature review found eight articles reporting on this so-called double crush syndrome of the L5 NR compression and distal peroneal nerve.

The underlying pathophysiologic mechanism of DCS remains unclear, and a real definition is lacking. Recent efforts to create more consensus resulted in four highly plausible mechanisms to explain this phenomenon: impaired axonal transport, distorted ion channel up- or downregulation, immune-inflammation of the dorsal root ganglia, or formation of a neuroma-in-continuity [30]. In addition, it has been suggested to rephrase it to multifocal neuropathy, as more than two distinct lesions on one nerve can occur simultaneously [14], and other etiologies than compression (crush) have been described as potential causes [35]. Despite the ongoing controversies, there seems to be an understanding among medical professionals that the phenomenon “double crush syndrome” to some extent exists, and treating physicians should be aware of this [13,30].

Current literature on DCS is predominantly on concomitant cervical radiculopathy and distal median or ulnar nerve entrapment, with reported incidences widely varying [19,36,37]. Only a few large series have been published on the occurrence of DCS of the lower extremities. In an electrodiagnostic study of 169 patients and 289 measured peroneal nerves, a strong association between proximal lumbosacral nerve root pathology and distal nerve compression syndromes was found [28]. Co-occurrence of lumbosacral radiculopathy and tarsal tunnel syndrome has also been reported [38].

Recently, a comparable retrospective study on DCS was published, in which 10 patients with active L5 radiculopathy and peroneal neuropathy based on EMG, NCS and imaging studies underwent decompression of the CPN [29]. All patients reported improved strength of the dorsiflexion, extensor hallucis longus, and ankle eversion after decompression. Pain improved in 33% of the patients with pre-operative pain and numbness improved in 56% of the patients (Table 2). Although the authors advocate CPN release as the first surgical treatment option when L5 radiculopathy and peroneal neuropathy co-exist, their results appear to be less beneficial towards this strategy than our findings. This can partly be explained by the different inclusion criteria. They only included patients with concomitant active L5 radiculopathy and active peroneal neuropathy using NCS and EMG (referred to by the authors as ‘pure’ DCS). In our study, patients were included with a suspicion of co-existing pathologies along their clinical course, and a variety of diagnostics have been performed at different time points to support that, mostly MRI and US. Even though this is a better reflection of clinical practice, technically, it does not prove an active L5 radiculopathy and active peroneal neuropathy at the same time and should be taken into account when interpreting our results. Nerve conduction studies and electromyography would in this case be ideal to demonstrate an active dual-lesion neuropathy and subsequent denervation (see also Figure 1). In 2023, a study of 13 patients with CPN entrapment and co-existing or a history of L5 radiculopathy also showed remarkable improvement in pain, dysesthesia and motor deficits after neurolysis of the CPN. However, compared to a group of patients after CPN decompression without L5 radiculopathy (nine patients), there was no statistically significant difference in outcome [20]. Furthermore, in a large series of 300 patients operated on for lumbar disk herniation, 3 were misinterpreted as having failed back surgery syndrome while having additional entrapment of crural peroneal branches. Subsequent neurolysis resulted in a successful outcome (not further specified) [21]. Several other case reports showed significant improvement of pain and, to a lesser extent, functional recovery of motor deficits when treated surgically [22,23] or conservatively [24,26] for common peroneal nerve entrapment after spinal surgery.

Due to our small sample size, study design, and clinical heterogeneity, it is not possible to give any treatment recommendations based on our results. Although newer techniques for spine surgery are becoming less invasive, decompression of CPN at the fibular head still remains a relatively simpler surgical procedure with fewer complications and shorter recovery time compared to spine surgery. Especially when compared to a posterior lumbar interbody fusion (PLIF) procedure, which is often performed in our center for a foraminal stenosis in case of a spondylolytic spondylolisthesis. Based on our findings and those of comparative studies [20,29], CPN compression should be strongly considered as a primary treatment option if DCS of the L5 nerve and CPN is suspected.

### 4.1. Clinical Implications

As mentioned before, two distinct groups could be identified in this cohort. The first group consisted of seven patients (50%) who were initially referred to our clinic for spinal surgery with symptoms and radiological findings fitting L5 NR pathology. However, their history and neurologic examination suggested concurrent CPN entrapment, which was confirmed by US or NCS/EMG. All of these patients had successful outcomes after CPN decompression. The other seven patients (group B) had been surgically (N = 5) or conservatively (N = 2) treated for L5 NR compression but had persisting or recurrent symptoms suggestive of CPN compression at the fibular head. In 85% of the patients in this group, additional CPN decompression resulted in almost complete or complete recovery of symptoms. 

The first group demonstrates the importance of accurate clinical examination and additional work-up if symptoms differ from typical L5 radiculopathy, e.g., pain is in the lower leg or sensorimotor deficits of the foot are more prominent. Specific muscle testing (such as inversion vs. eversion of the ankle or the assessment of hip adductor strength [39]) and the presence of Tinel’s sign [6,7,29,40] can help to differentiate between an L5 NR problem or distal peroneal neuropathy. The latter, however, was only positive in two of our seven patients, so the usefulness appeared to be limited in our cohort. The presence of back pain can be misleading. In a large series of patients referred for lumbar radiculopathy but in the end were diagnosed with a distal nerve entrapment, 49% of the patients also reported back pain [41]. In our series, this was even higher: 85% of the patients at initial presentation. Thus, the presence of back pain does not assume an NR problem as the cause of the leg symptoms. With regard to diagnostics, MRI remains the most important tool to assess L5 nerve root pathology; however, radiological NR compression can occur without radicular leg pain [42]. In our experience, US, NCS, and needle EMG are crucial in the workup to assess potential concurrent peroneal neuropathy [8,43]. In addition to assessing abnormal thickening of the CPN as a sign of compression [44,45,46], US can also detect other anatomical abnormalities (e.g., intraneural ganglion cysts) [47].

The second group in our cohort shows the importance of recognizing the possibility of peroneal neuropathy after surgically or conservatively treated L5 radiculopathy when there are persisting or recurrent symptoms. Especially if the radiation pattern is shifted to the lower leg or sensorimotor defects are more prone. If unrecognized, patients can be withheld adequate treatment (i.e., CPN decompression) and referred for other unnecessary treatments, e.g., selective nerve root injections, or even in some cases more extensive lumbar surgery and additional fusion, as was suggested by referring physicians. In the recent publication of Santangelo et al., there were also three patients (30%) who had lumbar surgery prior to CPN decompression. Unfortunately, no additional information is provided to make further comparisons.

The variety in presentation, diagnostic findings and treatment choices seen in our series resembles the daily clinical practice when dealing with (potential) co-existing dual compression of the same nerve, making it a very challenging clinical entity to deal with. Due to significant overlap in symptoms, patients’ history and examination can be misleading. Also, the sensitivity of specific tests of peripheral nerve disease, such as the presence of a Tinel’s sign, appears to be as low as is shown in our study. Despite this, we want to stress the importance of the patient’s history and thorough examination if patients are presented with apparent lumbosacral pathology. If radiating patterns are predominantly of the lower leg or when sensorimotor deficits of the foot are more prominent, peroneal neuropathy should be considered in the differential diagnosis, and diagnostic testing should be performed accordingly.

### 4.2. Limitation

The observational nature of the study resulted in clinical variety and different treatment strategies, making it difficult to address important clinical questions and provide solid recommendations. Despite most of the data being gathered prospectively, missing data still occurred. A very important limitation in this case is the lack of pre- and post-operative electrodiagnostic testing (NCS and EMG), while this is essential in assessing active concomitant proximal and distal nerve compression. In our center, however, US is preferred to diagnose CPN entrapment and to rule out any structural abnormalities (e.g., intraneural ganglion cysts), and since it was an observational study, no additional diagnostic testing could be performed for study purposes. In case the US was normal or to further differentiate between L5 radiculopathy and peroneal neuropathy, additional NCS/EMG was performed. If symptoms and US findings were fitting CPN entrapment at the fibular head, additional electrodiagnostic testing would not have altered the surgical decision-making. Furthermore, the main outcome in this study was the subjective improvement of pain. In future studies, it would be better to include a more objective pain assessment on the Visual Analog Scale as well as objective measurements of sensibility using, e.g., the Semmes–Weinstein monofilament test. Lastly, in continuation of the ongoing debate about how DCS should be defined and what it compels, one can argue whether the criteria for ‘pure’ DCS in our study are met, especially in the aforementioned second group. This does not detract from the fact that our study population is a good reflection of the daily clinical practice in which L5 NR and CPN pathology somewhere along their clinical course can co-exist, including all the challenges that come with managing these patients. Future research should be directed at refining the patient selection process by standardizing pre-operative clinical and diagnostic tests, including MRI for lumbar radiculopathy and both US and NCS/EMG for peripheral neuropathy if DCS is suspected. Additionally, a predefined set of patient-reported outcome measures should be collected to assess the outcome of the different treatment strategies.

## 5. Conclusions

Treating physicians should be aware that double crush syndrome of the L5 nerve root and common peroneal nerve at the fibular head can occur, and additional nerve conduction or ultrasound investigations are warranted when patients present with atypical radicular symptoms and predominant sensorimotor deficits of the lower leg, especially if persisting or recurring after spinal surgery. Adequate detection can prevent any delay in treatment, in this case, a less invasive peroneal release, and prevent unnecessary (additional) spinal surgery. The role of the neurosurgeon in this is pivotal. Being familiar with both conditions, the neurosurgeon can best weigh the risks and benefits of the (non)surgical treatment options for this complex problem.

## Figures and Tables

**Table 1:** Patient characteristics, diagnostics and outcome.

No.	Age	Sex	BMI	Side PN	Clinical Features			Pre-Operative Work Up					Previous Treatment L5 NR Compression	FU (mo)	Outcome After CPN Decompression		
					Clinical Symptoms	Back-Pain	Tinel’s Sign Fibula Head	NCS	EMG	MRI	US *				Likert Scale	Strength Improved	US **
											Right	Left					
**1**	48	M	30	L	Pain; loss of strength: DF MRC 4	No	absent	Low conduction velocity CPN over EDB and TA, low amp CMAP	Paraspinal normal, fib TA	Foraminal stenosis L5	-	37.4	-	9	Likert 2	No	17.5 (−)
**2**	49	F	31	R	Numbness; loss of strength: DF and Eversion MRC 4+	Yes	absent	No CMAP CPN over EDB, low amp CMAP over TA	Reinnervation TA	Lytic spondylolisthesis L5-S1, foraminal stenosis	13.0	12.9	-	2	Likert 1	Yes	-
**3**	67	M	25	R	Pain; numbness; loss of strength: DF MRC 3, EHL MRC 0	No	present	Not performed	Not performed	Bilateral recess stenosis, deg spondylolisthesis	12.1	12.0	-	3	Likert 2	Yes	-
**4**	52	F	29	R	Pain; numbness	Yes	absent	Not performed	Not performed	Spondylolysis, herniated disk L4-5	12.1	12.0	-	2	Likert 2	-	-
**5**	56	M	28	R	Pain; numbness	Yes	absent	Not performed	Not performed	Foraminal stenosis L5	19.0	-	-	2	Likert 1	-	-
**6**	69	M	24	R	Pain; numbness	Yes	present	Not performed	Not performed	Lateral recess stenosis L4-L5	14.0	-	-	7	Likert 2	-	-
**7**	74	F	31	R	Pain; numbness; loss of strength: DF MRC 4	Yes	present	Low conduction velocity CPN, low amp CMAP	Evidence of PN compression, not supportive for L5 NR	Lateral recess stenosis L4-L5	10.9	-	NA (secondary decompression L4-L5)	13	Likert 1	NR	12.7 (+)
**8**	61	M	NR	L	Loss of strength: DF MRC 4, eversion MRC 4	Yes	absent	Low CMAP	Net performed	Herniated disk L4-5 and L5-S1	-	16	Decompression L4-5 and L5-S1 left	4	Likert 2	Yes	-
**9**	63	M	34	L	Numbness	Yes	absent	Normal conduction velocity and CMAP CPN	Reinnervation of peroneus longus	stenosis L4-L5 with instability	15.9	14.1	PLIF L4-L5	1	Likert 1	-	-
**10**	50	M	34	R, L	Pain; loss of strength: DF MRC 4, eversion MRC 4	Yes	present	Normal conduction velocity and CMAP CPN	Not performed	lytic spondylolisthesis L5-S1	12.1	15	PLIF L5-s1	9	Likert 2	Yes	14.5 (=)
**11**	63	M	27	L	Loss of strength: DF MRC 3, eversion MRC 3	yes	absent	Normal conduction velocity and CMAP CPN	Paraspinal normal, reinnervation TA	lateral recess stenosis L4-L5	15.7	15.1	Decompression L4-L5	4	Likert 2	Yes	-
**12**	43	M	25	L	Loss of strength: DF MRC 1	yes	absent	No CMAP CPN over EDB and TA	Denervation TA pos waves and fib	stenosis L4-L5, herniated disk l3-4	12.1	14.4	Decompression L4-L5, discectomy L3-4	14	Likert 2	No	-
**13**	42	M	25	L	Pain; loss of strength: DF MRC 4, eversion MRC 4	yes	absent	Low CMAP CPN over EDB and TA	Polyfascicular L5 paraspinal	no recurrent herniated disk	-	16	Conservative treatment	11	Likert 3	Yes	-
**14**	65	M	34	L	Pain; loss of strength: DF MRC DF 4	yes	absent	Not performed	Not performed	Lateral herniated disk l4-5 left	-	23	Conservative treatment	13	Likert 2	Yes	7.5 (−)

F = female; M = male; L = left; R = right; BMI = Body Mass Index; CPN = common peroneal nerve; NR = nerve root; DF = dorsiflexion; EHL = extensor hallucis longus; MRC = Medical Research Council scale; NCS = nerve conduction studies; EMG = electromyography; MRI = magnetic resonance imaging; US = ultrasound; TA = tibialis anterior muscle; EDB = extensor digitorum brevis muscle; CMAP = compound muscle action potential; fib = fibrillations; L = lumbar; PLIF = posterior lumbar interbody fusion; FU = follow-up; mo = months; NR = not reported; * reported are the cross-sectional area (CSA) in mm^2^ of the peroneal nerve at the fibula head; in italics are the asymptomatic sides. ** If during the post-operative follow-up US was performed, (+) was used if CSA was increased compared to pre-operatively, (−) if it was reduced, or (=) if there was no big difference.

**Table 2:** Review of literature.

Author and Year	Type of Study	No of Patients	“Proximal Event”	“Distal Event”	(Surgical) Treatment	Outcome
Crotti, 2005 [21]	Case series	3/300 (1%)	L5 nerve root compression (N = 300)	Entrapment of crural branches of peroneal nerve (N = 3)	First: Removal of lumbar disk hernia Second: Neurolysis of crural branches of PN	Not specified
Reife, 2013 [24]	Case report	1	L5 nerve root compression	Entrapment of the CPNat the fibula head	Decompression pf L5 NR by hemilaminectomy with removal of herniated disk. Complicated by CSF fistula. Conservative treatment of peroneal neuropathy	Complete recovery
Ang, 2014 [22]	Case report	1	L5 nerve root compression	Entrapment of the sup peroneal nerve in the lateral calf	First: Decompression of the L5 NR lateral recess Second: Neurolysis of the superior PN	12 months after second surgery complete recovery
Park, 2019 [26]	Case report	1	L5 nerve root compression	Entrapment of the CPN at the fibula head (intraneural ganglion cyst)	First: Conservative treatment of L4-5 herniated disk Second: Ultrasound aspiration of cyst (refused surgical treatment)	Slight motor function improvement, improved tingling sensation, no difference in numbness. EMG indicated regeneration of PN
Maejima, 2021 [23]	Case report	1	L5 nerve root compression	Entrapment of theCPN at the fibula head	First: Posterior lumbar interbody fusion l5-s1 Second: Neurolysis of the CPN	Reduced pain and partially improved motor function. 46.2% improvement in JOA score
Shields, 2022 [25]	Case report	2	L5 nerve root compression	Entrapment of the CPN at the fibula head (1 case of intraneural ganglion cyst)	In one case, first dissection of CPN and drainage of cysts; secondly, bilateral L3-S1 laminectomy, facetectomy and foraminotomy, and L5-S1 fusion; in the other case, no surgery has been performed	Surgical case: Significant improvement Conservative case: Complete recovery of function
Ishii, 2023 [20]	Case series	22 (25 limbs)	L5 nerve root compression	Entrapment of the CPN at the fibula head	Two groups were retrospectively compared; all patients had neurolysis of the CPN at the fibula head: Group R (13 pts/15 limbs): additional history of L5 radiculopathy Group O (9 pts/10 limbs): without L5 radiculopathy	No statistical difference in outcomes between the groups
Santangelo, 2025 [29]	Case series	10	L5 radiculopathy	Entrapment of CPN at the fibular head	All pts underwent CPN decompression at the fibular head: Three pts (30%) had prior lumbar spine surgery but still had active L5 radiculopathy when CPN decompression was performed	All showed improved strength; pain below the knee improved in 33% (2 out of 6 pts), and numbness in 56% (5 out of 9 pts)

No = number; NR = nerve root; PN = peroneal nerve; CPN = common peroneal nerve; CSF = cerebrospinal fluid; JOA = Japanese orthopedic association score; pts = patients.

## Data Availability

No data is available due to privacy or ethical restrictions.

## Abbreviations

The following abbreviations are used in this manuscript: DCS = double crush syndrome; NR: nerve root; CPN = common peroneal nerve; L5 = 5th lumbar vertebra; US = ultrasound; NCS = nerve conduction studies; EMG = electromyography; MRC scale = Medical Research Council scale; CSA = cross-sectional area.
